# High expression of FABP4 and FABP6 in patients with colorectal cancer

**DOI:** 10.1186/s12957-019-1714-5

**Published:** 2019-10-24

**Authors:** Yaqin Zhang, Xiaotong Zhao, Lili Deng, Xueting Li, Ganbiao Wang, Yongxing Li, Mingwei Chen

**Affiliations:** 1 Department of Endocrinology, the First Affiliated Hospital of Anhui Medical University, 210 JiXi Road, Hefei, 230032 People’s Republic of China; 2 Department of General Surgery, the First Affiliated Hospital of Anhui Medical University, Hefei, People’s Republic of China; 3 Institute of Diabetes Prevention and Control, Academy of Traditional Chinese Medicine, Hefei, 230032 People’s Republic of China

**Keywords:** Colorectal cancer, FABP4, FABP6, Biomarkers

## Abstract

**Objective:**

To explore the relationship between FABP4 and FABP6 expression and the pathogenesis of colorectal cancer (CRC) and their potential as biomarkers in the diagnosis of CRC.

**Methods:**

In total, 100 CRC patients and 100 controls were enrolled. The serum levels of FABP4 and FABP6 were detected by enzyme-linked immunosorbent assay (ELISA) before and 2 weeks after radical resection of CRC. The protein expressions of FABP4 and FABP6 were observed in colorectal tumor tissues and adjacent tissues by immunohistochemistry and western blot, respectively. The diagnostic performance of FABP4 and FABP6 in patients with CRC was evaluated by receiver operating characteristic (ROC) curve analysis.

**Results:**

The serum levels of FABP4 and FABP6 in patients with CRC were higher than the levels in the controls before surgery (*P* < 0.001), and significantly decreased at 2 weeks after operation (*P* < 0.001). Immunohistochemistry showed that FABP4 and FABP6 were mainly distributed in the cytoplasm of human colorectal tumor tissues, and only a small amount distributed in adjacent tissues. Western blot revealed that the protein expressions of FABP4 and FABP6 were significantly higher in tumor tissues than in adjacent tissues (*P* < 0.001, *P* = 0.002, respectively). Tumors with high and low FABP4 and FABP6 expression have no significant correlation in tumor size, tumor site, distant organ and lymph node metastasis, histologic grade, lymphatic permeation, neurological invasion, vascular invasion, and Duke’s and TNM classification. Multivariate logistic regression analysis showed that FABP4 and FABP6 were independent risk factors for CRC (adjusted odds ratio 1.916; 95%CI 1.340–2.492; *P <* 0.001; adjusted odds ratio 2.162; 95%CI 1.046, 1.078); *P <* 0.001, respectively). In discriminating CRC from the normal control, the optimal sensitivity of FABP4 and FABP6 were 93.20% (95%CI 87.8–96.7) and 83.70% (95%CI 76.7–89.3), respectively, while the optimal specificity of FABP4 and FABP6 were 48.8% (95%CI 39.8–57.9) and 58.4% (95%CI 49.2–67.1), respectively. When combined detection of serum carcinoembryonic (CEA) and FABP4 and FABP6, the optimal sensitivity and specificity were 61.33% (95%CI 53.0–69.2) and 79.82% (95%CI 71.3–86.8), respectively.

**Conclusion:**

Increased expression of FABP4 and FABP6 not only were strong risk factors for the development of CRC but could also represent a potential biomarker for CRC diagnosis in Chinese patients. Combined detection of CEA with FABP4 and FABP6 could improve the diagnostic efficacy of CRC.

## Introduction

Worldwide, the incidence of colorectal cancer (CRC) ranks third in malignant tumors and ranks second in terms of mortality [1]. In 2014, ~ 65,000 women and 71,830 men were diagnosed with CRC [2], and it is estimated that more than 50% of CRC patients will die from the disease [3]. It is generally believed that the significant increase in the incidence of CRC in both developed and developing countries may be closely related to the aging of the population, unhealthy eating habits (e.g., excessive intake of animal fat, insufficient intake of cellulose), smoking, lack of physical exercise, and obesity [4].

Fatty acid-binding proteins (FABPs) are a group of low-molecular-weight proteins involved in the transport of long-chain bioactive fatty acids in cells. Since FABPs were discovered in 1972, 12 different types (FABP1-12) have been confirmed, nine (FABP1–FABP9) are expressed in humans. According to the current literature, FABPs are expressed differently in different types of cancer or in different cell lines of the same cancer. FABP4 is mainly expressed in differentiated adipocytes and macrophages [5], and previous studies have focused on its association with metabolic syndrome and its related components, especially obesity [6]. As we all know, obesity is a risk factor for CRC, whereas the role of FABP4 and its underlying molecular mechanism in tumorigenesis of CRC have not been elucidated. Nieman et al. have detected FABP4 in ovarian cancer cells at the interface of the adipocyte-tumor cell [7], implying that FABP4 may be secreted from adipocytes and transferred to tumor cells. In addition, several investigations showed that FABP4 could promote cell growth and metastasis in breast and prostate cancers [8, 9]. These studies suggested that FABP4 might be involved in the development and progression of carcinoma.

FABP6 is highly expressed in the ileum and is an intracellular transporter of bile acids in ileal epithelial cells, which helps catalyze and metabolize cholesterol. Fecal bile acid concentrations, particularly secondary bile acids, are known to be higher in colon adenomas or CRC patients [8–11], whereas in vitro, bile acids induce the colon cancer cell line’s (Caco-2) high expression of FABP6 [12]. Ohmachi et al. has identified FABP6 is overexpressed in CRC and plays an important role in the early canceration of CRC [13]. Although many researchers have made great efforts to clarify these mechanisms of FABP6 in regulating CRC, it still had not been well elucidated.

Recent studies have shown that FABPs may play an important role in cells proliferation [7, 14], suggesting that the changes in the expression of FABPs in individuals during tumor progression may contribute to the development of tumors [15], and the expression of FABPs in different tumors may also serve as diagnostic markers and/or new therapeutic targets [16], such as gastric cancer and FABP3 [17], and prostate cancer and FABP5 [18]. Therefore, FABPs are expected to be candidates for biomarkers in the diagnosis or prognosis of cancer.

So far, fewer clinical studies concerning the association between FABP4 and FABP6 and CRC are reported [13, 19, 20]. This article aims to explore the relationship between FABP4 and FABP6 and the pathogenesis of CRC and its potential as a serum biomarker with potential value in the diagnosis and prognosis of CRC. The main contents are as follows: (1) The serum levels of FABP4 and FABP6 were detected by enzyme-linked immunosorbent assay (ELISA) before and 2 weeks after radical resection of CRC; (2) the protein expressions of FABP4 and FABP6 were observed in colorectal tumor tissues and adjacent tissues by immunohistochemistry and western blot, respectively; (3) the association between FABP4 and FABP6 levels and the clinicopathological variables of CRC patients were investigated; (4) multivariate logistic regression analysis was applied to explore whether FABP4 and FABP6 were independent risk factors for CRC; and (5) the diagnostic performance of FABP4 and FABP6 in patients with CRC were evaluated by receiver operating characteristic (ROC) curve analysis, especially, intending to probe whether combined detection of FABP4 and FABP6 with conventional blood-borne biomarker such as carcinoembryonic (CEA) and carbohydrate antigen 19-9 (CA199) could improve the diagnostic efficacy (sensitivity and specificity) of CRC. To the best of our knowledge, this is the first study to systematically evaluate the role of FABP4 and FABP6 on the development of CRC and their potential as biomarkers in the diagnosis of CRC.

## Materials and methods

### Study participants

In all, 100 consecutive Chinese patients of Han nationality with pathologically or biopsy-diagnosed CRC who had not received surgery, radiotherapy, or chemotherapy, between September 2017 and December 2018, at the First Affiliated Hospital of Anhui Medical University, were enrolled in this study. Of the 100 patients, 38 had colon cancer, and the remainder had rectal cancer. The control subjects were 100 consecutive Chinese subjects of Han nationality without colorectal polyp or inflammatory bowel disease, who underwent a total colonoscopy after a voluntary health check-up or occult fecal blood loss at the same hospital. Each control was matched with one case for gender, age, BMI and time to the admission of the corresponding case (± 1 month). The exclusion criteria were patients with familial adenomatous polyposis, hereditary non-polyposis CRC, previous gastrointestinal tract surgery, inflammatory bowel disease, serious liver and renal dysfunction, and acute and chronic infectious disease. Patients with CRC were treated with radical resection of CRC, and TNM staging was performed on the post-operative pathology results according to the criteria (NCCN, Colon Cancer Guidelines, 2017) to assess the extent of cancer invasion. According to tumor localization, samples were classified as “right-sided” (localized in the caecum or in the ascending or transverse colon) and “left-sided” (set in the descendant or sigmoid colon or in the rectum). According to tumor size, two groups were identified: the first comprised tumors ≤ 5 cm in size, and the second consisted of tumors > 5 cm in size. Local invasion was also classified into two groups, pT1-T2 and pT3-T4, respectively. Moreover, cases were subdivided into two groups based on their histological grade, the first group comprising grade 1 and grade 2 cases, and the second group consisting of grade 3 adenocarcinomas. This study was approved by the Ethics Committee of the First Affiliated Hospital of Anhui Medical University in March 2017 and has been registered in the Clinical Drug Evaluation Center of Anhui Medical University as CDEC000002712. Statement of consent to participate in this study was obtained in advance from all participants.

All subjects fasted for 10 h and then were measured for height, weight, waist circumference (WC), hip circumference, diastolic blood pressure (DBP), and systolic blood pressure (SBP). Body mass index (BMI) and waist-to-hip ratio (WHR) were calculated, and a questionnaire about their smoking habits, alcohol intake, medications (e.g., antihypertension/antihyperlipidemic drug use and aspirin) and family history of CRC were completed. Any person who had smoked an average of more than five cigarettes every day for a year was defined as a regular smoker, whereas those who had stopped smoking for more than a year previously were counted as ex-smokers. Any person who drank an average of more than 25 g of alcohol daily for 1 year was defined as a regular alcohol drinker. A family history of CRC was defined as a first-degree relative with CRC, respectively [21].

### Laboratory procedures

Two hundred subjects had 8 ml of blood drawn from the cubital vein in the morning after fasting for 10 h. Of the 100 patients with CRC, 8 ml of blood was again taken under the same conditions after surgery (2 weeks). The collected blood samples were centrifuged, and the serum was aspirated and stored in a − 80 °C refrigerator until analysis. Blood glucose and blood lipids were measured using an automatic biochemical analyzer (MODULE P800, Roche, Switzerland). Among them, fasting plasma glucose (FPG) was detected by glucose oxidase method. Triacylglycerol (TG), total cholesterol (TC), high-density lipoprotein cholesterol (HDL-C), and low-density lipoprotein cholesterol (LDL-C) were detected by enzymatic method. CEA and CA19-9 were detected by electrochemiluminescence (Roche, Cobas 601). The serum levels of FABP4 and FABP6 were measured by ELISA kits based on the double antibody sandwich technique (Wuhan Gene Beauty Technology Co., Ltd., model JYM1843Hu, JYM18424Hu), and the sensitivity of interval assay was 0.1 pg/ml. Intra- and inter-assay variability should be less than 9% and 15%, respectively. Standards, controls, and samples were evaluated at a wavelength of 450 nm. Three measurements were made in a single experiment and determined by comparing the absorbance (OD value) of the sample to a standard curve. Referring to previous research [13], in the present study, based on the median of FABP4 and FABP6 levels of CRC patients, CRC patients were divided into 2 subgroups: high-expression group (above the median) and low-expression group (below the median). In addition, intraoperative cancer tissues and adjacent tissues (> 5 cm from cancer tissue) were collected in patients with CRC, in duplicate, approximately 2 cm × 1 cm × 1 cm, one in a liquid nitrogen tank and the other in a liquid nitrogen tank fixed in 10% formalin.

### Immunohistochemical staining for FABP4 and FABP6

The embedded tissue was trimmed, the slicer was placed in a 4-μm section, and the oven was dewaxed and hydrated overnight. Three times xylene (Shanghai Susie) and 100%–95%–80% ethanol were passed in sequence for 15 min. The slices were placed together with the staining rack into the beaker and rinsed with tap water slowly until the slices were clean and transparent. A pressure cooker was used to hold 2 L of double distilled water, and 40 ml of pH 8.0 ethylenediaminetetraacetic acid (EDTA) repair solution was added and heated to boiling on the induction cooker. Then, the slices were put together with the dyeing rack into the repair solution, fixed after boiling for 2 min, and allowed to cool naturally. Next, 3% H_2_O_2_ was added to the tissue and incubated (room temperature, 20 min). The samples were washed three times with phosphate-buffered saline (PBS), and the sections were dried. An appropriate amount (~ 100 μl) of primary antibody (FABP4: murine monoclonal antibody; FABP6: rabbit polyclonal antibody, Bioss, China) was added and incubated (37 °C, 60 min). After rinsing, 100 μl or an appropriate amount of secondary antibody (general-purpose secondary antibody kit, horseradish peroxidase-labeled goat anti-mouse/rabbit IgG, Zsbio, China) was added dropwise and incubated again (room temperature, 20 min). After washing, add diaminobenzidine (DAB) coloring agent was added, the color development time under the microscope (CX43, OLYMPUS) was controlled (there is a positive termination color development), and the color development was stopped and rinsed with distilled water. Finally, the stained sections were stained for 1 min in hematoxylin staining solution, differentiated with 1% hydrochloric acid alcohol for several seconds, and washed again. After washing, the expression and distribution of FABP4 and FABP6 were observed under a microscope (in accordance with the antibody specification, FABP4 and FABP6 were stained with DAB, and brown was positively observed under a microscope). The average optical density value (ImagePro Plus 6.0 software analysis image) was calculated by immunohistochemical morphological analysis software to quantitatively compare the difference between the protein expressed by the positive cells in the cancer tissue and the adjacent tissues. In addition, we deal positive control tissues sections (confirmed homologous tissue sections containing FABP4 and FABP6 antigens) and experimental tissues sections of this study with the same treatment and immunostaining to confirm the effectiveness of the immunohistochemical staining procedure and to exclude false negatives.

### Western blot analysis of FABP4 and FABP6

Total protein was isolated from the ground tissue samples using radioimmunoprecipitation assay (RIPA) buffer. Briefly, a 100-mg sample was mechanically comminuted and resuspended in 1 ml RIPA buffer (100 mg tissue/ml). The resuspended sample was sonicated on ice, the insoluble matter was removed by centrifugation (12,000×*g*, 10 °C for 10 min), and the supernatant was retained. Protein samples were separated by 10% sodium dodecylsulfate-polyacrylamide gel electrophoresis (SDS-PAGE) and transferred to a nitrocellulose membrane. After blocking 5% skim milk in Tris-buffered saline containing 0.05% Tween-20 (TBS-T) for 2 h, the membrane was incubated with primary antibody against FABP4 and FABP6 at room temperature overnight (FABP4: murine monoclonal antibody; FABP6: rabbit polyclonal antibody, Bioss, China); then, the secondary antibody was added and incubated at room temperature for 60 min (general-purpose secondary antibody kit, horseradish peroxidase-labeled goat anti-mouse/rabbit IgG, Zsbio, China) and washed. Finally, antibody detection was performed by ECL chemiluminescence (Thermo, USA).

### Statistical analysis

Excel 2013 was used for data aggregation, and the SPSS statistical software package and Medcalc 15.2 software of Windows vers.17.0 (SPSS, Chicago, IL, USA) were used for statistical analysis. Data were expressed as mean ± SD ($\overline{x}$ ± s). Differences in age, sex, BMI, WHR, SBP, DBP, FPG, TCH, TG, LDL-C, HDL-C, FABP4, FABP6, CEA, CA199, life style and personal and family medical history between patients and controls, the association of the localization and size of the tumor, and the histological grade as well as the clinical and pathological stage and serum FABP4 and FABP6 were assessed by *χ*^2^ test or by a one-way ANOVA analysis. Paired *t* test compared the change in pre- and post-operative BMI, FABP4, and FABP6. Spearman correlation coefficients were used to evaluate the correlations between serum FABP4 and FABP6 levels and other variables separately for controls and cases. To measure the associations between FABP4, FABP6 and the other variables with the risk of CRC, we calculated the adjusted odd ratios (OR) and their 95%CI using a conditional logistic regression model. In the logistic regression analysis, FABP4 and FABP6 and other variables were all analyzed as categorical variables and were classified into two categories based on the cutoff value, and potential confounding factors were adjusted. ROC curves were established to explore if FABP4 and FABP6 could be potential biomarkers for CRC. The optimal sensitivity and specificity from ROC curves were determined by commonly used methods [22]. All *P* values are two-sided and less than 0.05 were considered statistically significant.

## Results

### Comparison of clinical parameters and biochemical indicators between CRC group and control group

**Fig. 1:**
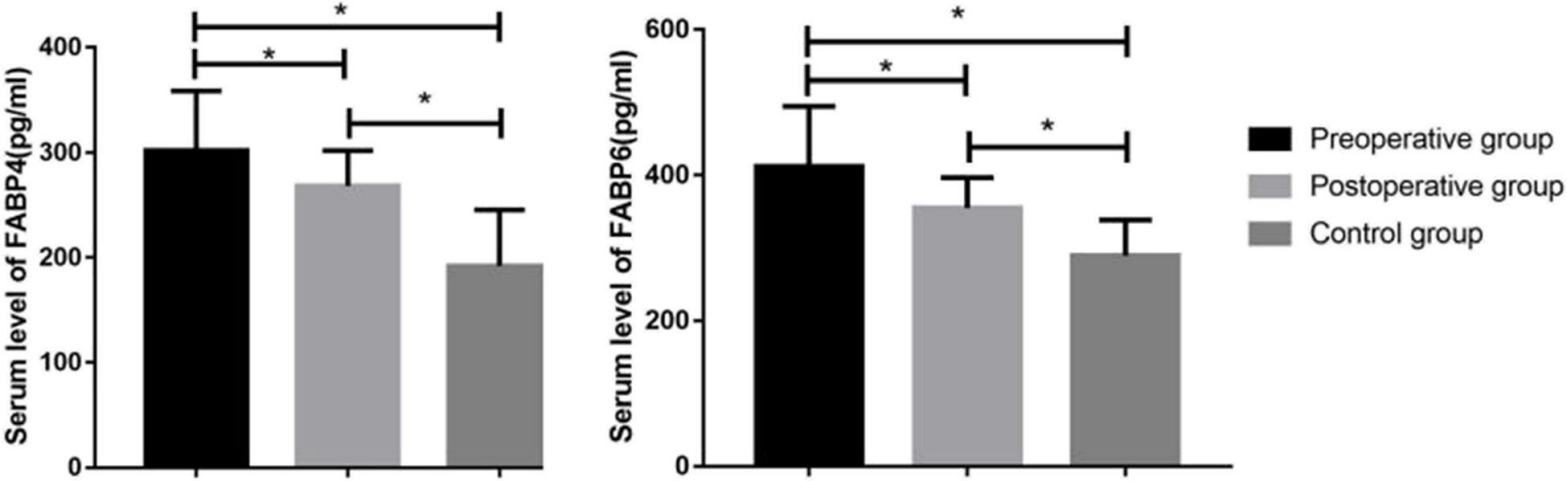
Comparison of serum levels of FABP4 and FABP6 between CRC group (including preoperation and postoperation) and control group. FABP4, fatty acid-binding proteins 4; FABP6, fatty acid-binding proteins 6. ^*^P < 0.001

There were no significant differences in age, sex, BMI, WC, WHR, BP, TG, FPG, the distribution of the numbers of current smokers, ex-smokers, habitual alcohol drinkers, habitual NSAID users, and diabetes between the CRC group before surgery and the control group. However, in the CRC group before surgery, TCH (*P* = 0.003), LDL-C (*P* = 0.001), FABP4 (*P* < 0.001), FABP6 (*P* < 0.001), CEA (*P =* 0.001), CA19-9 (*P* = 0.004), and the distribution of the numbers of family history of CRC (*P* = 0.03) were all higher, but HDL-C was (*P =* 0.006) lower compared with the control group. In addition, compared with preoperative levels, the patients’ BMI and WC (2 weeks after surgery) decreased slightly, but there were no statistical difference. However, the serum levels of FABP4 and FABP6 were significantly reduced (*P* < 0.001, *P* < 0.001, respectively); the decrease amplitude was 11.3% and 13.8%, respectively, whereas, it still higher than those in the control group (*P* < 0.001, *P* < 0.001, respectively). Moreover, in consistence with the changes of the serum levels of FABP4 and FABP6, the serum levels of CEA and CA-199 were all significantly decreased after surgery in the CRC group (*P* = 0.029, *P* = 0.048, respectively) (Table 1, Fig. 1).

**Table 1:** Comparison of clinical parameters and biochemical indicators between CRC group and control group [($\overline{x}$ ± s, *n* (%)]

Variable	CRC group (*n* = 100)		*P*^*a*^ value	Control group (*n* = 100)	*P*^*b*^ value
	Before surgery	After surgery			
Age (years)	55.50 ± 8.87	55.50 ± 8.87	NS	53.31 ± 10.58	NS
Sex					
Male	64 (64)	64 (64)	NS	36 (36)	NS
Female	53 (53)	53 (53)		47 (47)	
Smokers					
Current	21 (21)	21 (21)	NS	30 (30)	NS
Ex	10 (10)	10 (10)	NS	7 (7)	NS
Alcohol	40 (40)	40 (40)	NS	33 (33)	NS
NSAIDs	16 (16)	16 (16)	NS	10 (10)	NS
Diabetes	6	6	NS	5	NS
Family history of CRC	7 (7)	7 (7)	NS	1 (1)	0.030
BMI (kg/m^2^)	23.49 ± 2.73	23.42 ± 2.66	NS	22.81 ± 2.78	NS
WC (cm)	84.8 ± 8.6	84.6 ± 8.7	NS	83.5 ± 7.5	0.236
WHR	0.89 ± 0.07	0.89 ± 0.09	NS	0.87 ± 0.07	0.043
SBP (mmHg)	127 ± 16	126 ± 17	NS	128 ± 20	0.940
DBP (mmHg)	79 ± 11	80 ± 12	NS	82 ± 13	0.055
TCH (mmol/L)	4.77 ± 0.92	4.79 ± 0.89	NS	4.37 ± 0.90	0.003
TG (mmol/L)	1.59 ± 0.95	1.57 ± 0.87	NS	1.46 ± 0.80	0.287
LDL-C (mmol/L)	2.89 ± 0.97	2.88 ± 0.93	NS	2.53 ± 0.50	0.001
HDL-C (mmol/L)	1.17 ± 0.49	1.16 ± 0.52	NS	1.65 ± 0.14	0.006
FPG (mmol/L)	6.17 ± 4.45	6.34 ± 4.72	NS	5.50 ± 1.16	NS
FABP4 (pg/ml)	302.24 ± 56.58	268.08 ± 33.92	< 0.001	191.97 ± 53.49	< 0.001
FABP6 (pg/ml)	411.86 ± 83.25	354.64 ± 41.79	< 0.001	289.66 ± 48.57	< 0.001
CEA (ng/ml)	8.88 ± 1.26	6.72 ± 1.32	0.029	5.08 ± 0.81	0.001
CA-199 (U/ml)	14.50 ± 3.39	12.40 ± 4.36	0.048	11.02 ± 2.41	0.004

*Abbreviations*: *NSAID* non-steroid anti-inflammatory drug, *BMI* body mass index, *SBP* systolic blood pressure, *DBP* diastolic blood pressure, *WC* waist circumference, *WHR* waist:hip ratio, *TCH* total cholesterol, *TG* triglyceride, *LDL-C* low-density lipoprotein cholesterol, *HDL-C* high-density lipoprotein cholesterol, *FPG* fasting plasma glucose, *FABP4* fatty acid-binding proteins 4, *FABP6* fatty acid-binding proteins 6, *CEA* carcinoembryonic, *CA19-9* carbohydrate antigen 19-9, *NS* non-significant Intergroup comparisions, *P*^*a*^ after surgery vs before surgery, *P*^*b*^ control group vs CRC group before surgery)

### The protein expression and localization of FABP4 and FABP6 in CRC tissues and adjacent tissues

**Fig. 2:**
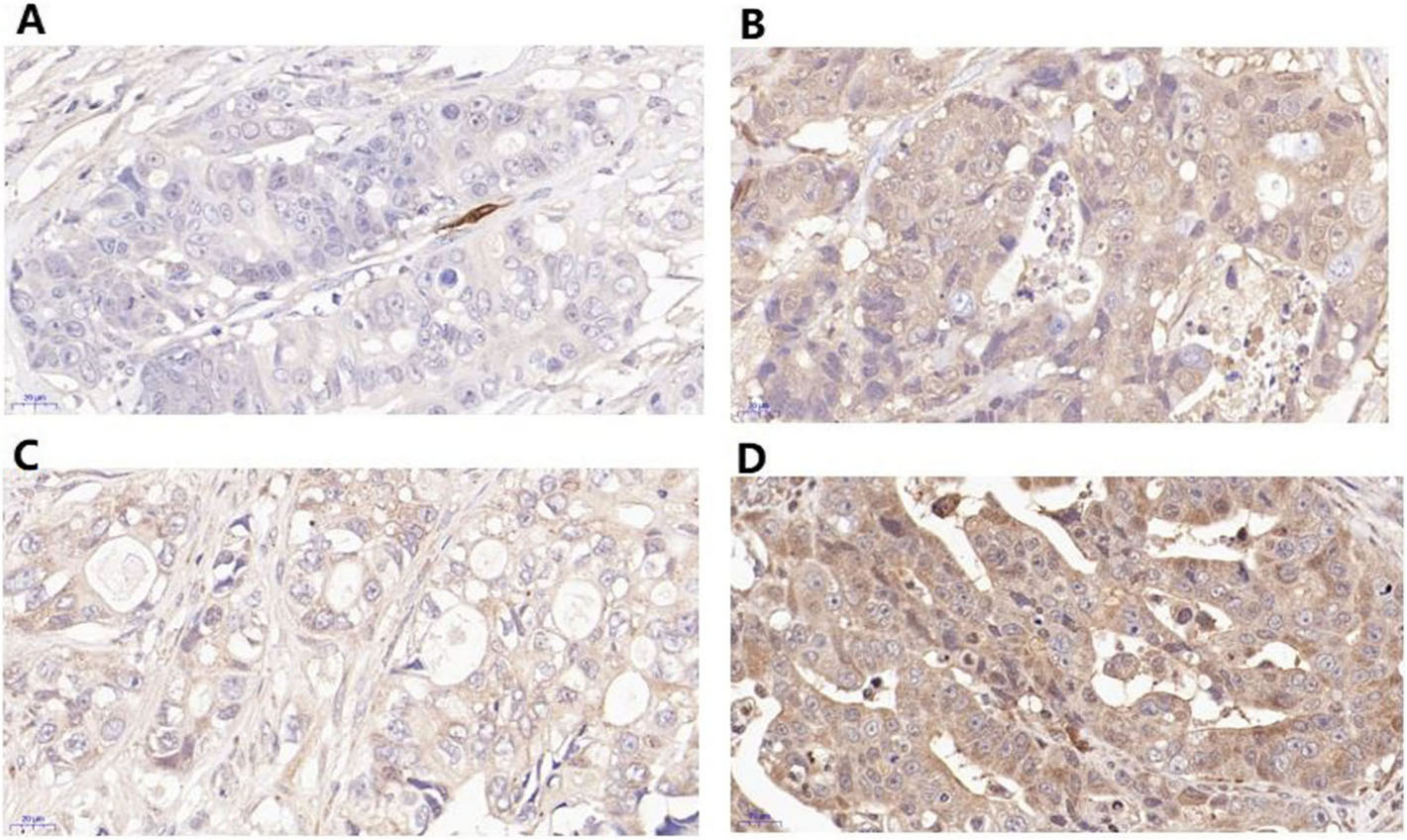
The comparison of the protein expressions of FABP4 and FABP6 between tumor tissues and adjacent tissues by IHC. The majority of the stain was observed in cancer cells. The average optical density of the colorectal tumor tissue sections was higher than that of the adjacent tissues (0.827 ± 0.114 vs 0.195 ± 0.025, *P* < 0.001). **a** Adjacent tissues FABP4. **b** Tumor tissues FABP4. **c** Adjacent tissues FABP6. **d** Tumor tissues FABP6

After immunohistochemical staining of the cancer tissue sections, it was observed under microscope that FABP4 and FABP6 were brown after staining, the positive sites were mainly distributed in cytoplasm of cells from human colorectal tumor tissues (FABP4: Fig. 2b, FABP6: Fig. 2d), and only a small amount was distributed in adjacent tissues (FABP4: Fig. 2a, FABP6: Fig. 2c).

**Fig. 3:**
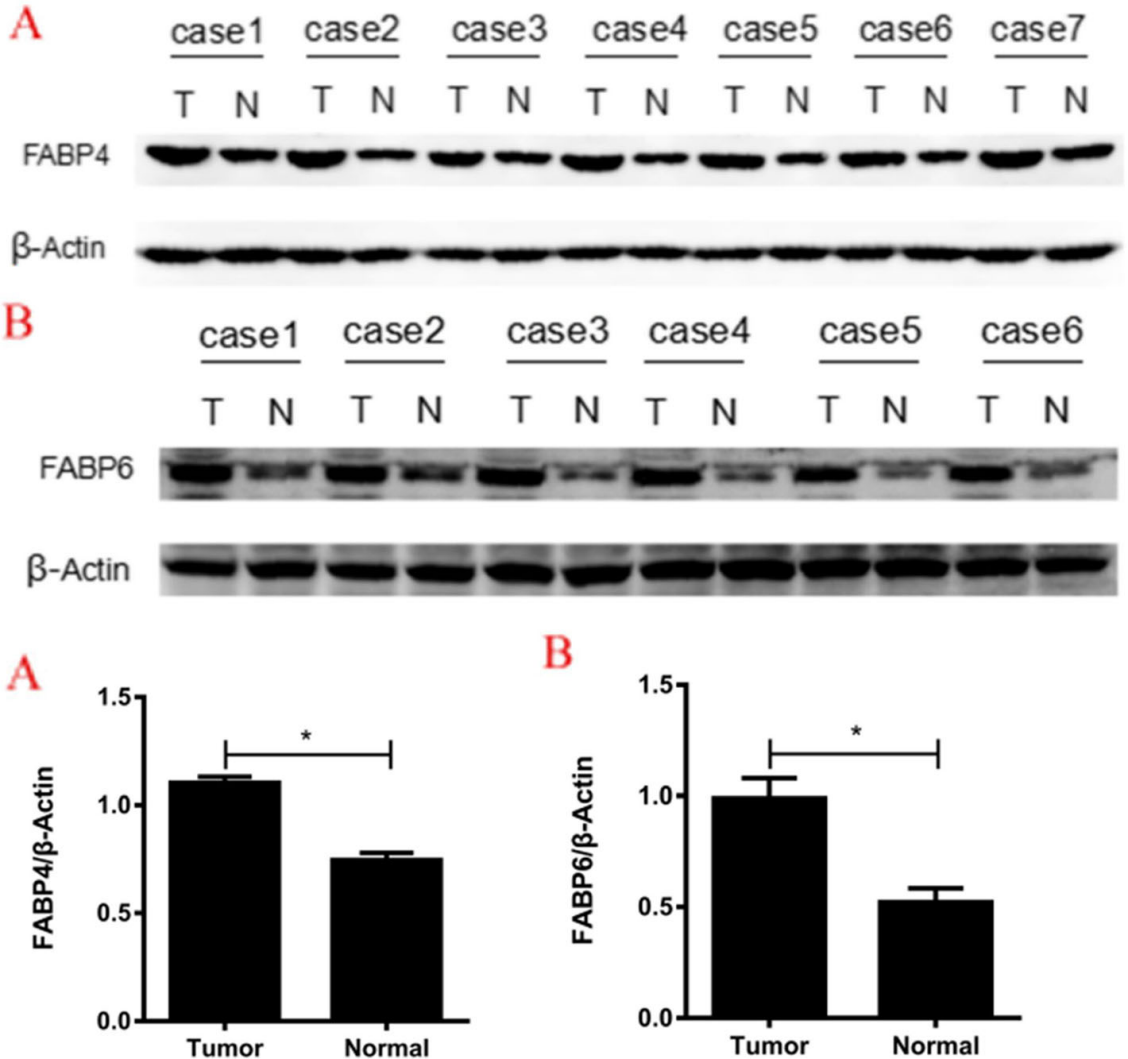
The comparison of the protein expressions of FABP4 and FABP6 between tumor (T) and adjacent (N) tissues by Western blot analysis.**a** FABP4. **b** FABP6

Western blot analysis showed that the protein expression levels of FABP4 and FABP6 in colorectal tumor tissues were higher than those in adjacent tissues (FABP4: 1.103 ± 0.529 vs 0.746 ± 0.296, *P* < 0.001; FABP6: 0.988 ± 0.225 vs 0.521 ± 0.156, *P* = 0.002) (Fig. 3).

### Relationship between the serum levels of FABP4 and FABP6 and clinicopathological features

To investigate the clinical significance of FABP4 and FABP6 expression, the serum FABP4 and FABP6 levels were categorized into low (< the median) or high (≥ the median) according to a cutoff value calculated from the median of the values observed in the CRC subjects. Tumors with high and low FABP4 and FABP6 expression have no significant correlation in tumor size (*P* = 0.838, *P* = 0.838, respectively), tumor site (*P* = 0.989, *P* = 0.716, respectively), distant organ and lymph node metastasis (*P* = 0.674, *P* = 0.674, respectively), histologic grade (*P* = 0.887, *P* = 0.748, respectively), lymphatic permeation (*P* = 0.418, *P* = 0.545, respectively), neurological invasion (*P* = 0.410, *P* = 0.317, respectively), vascular invasion (*P* = 0.689, *P* = 0.137, respectively), and Duke’s (*P* = 0.835, *P* = 0.209, respectively) and TNM classification (*P* = 0.953, *P* = 0.443, respectively).(Table 2).

**Table 2:** Relationship between the serum FABP4 and FABP6 levels and clinicopathologic features

Variable	FABP4		*P* value	FABP6		*P* value
	High expression (*n* = 50)	Low expression (*n* = 50)		High expression (*n* = 50)	Low expression (*n* = 50)	
Age	56.80 ± 8.25	54.0 ± 9.36	0.144	56.16 ± 9.10	54.84 ± 8.68	0.460
Sex						
Male	24	29	0.316	28	25	0.548
Female	26	21		22	25	
Tumor size (cm)						
≤ 5	30	31	0.838	31	30	0.838
> 5	20	19		19	20	
Tumor site^a^						
Left colon	11	5	0.989	10	6	0.716
Right colon	8	5		7	6	
Distant organ and lymph node metastasis						
Present	4	2	0.674	2	4	0.674
Absent	46	48		48	46	
Histologic grade^b^						
Poorly	10	12	0.887	12	10	0.748
Moderately	35	33		34	34	
Well	5	5		4	6	
Dukes classification^b^						
A+B	23	24	0.835	26	21	0.209
C+D	23	22		19	26	
TNM classification						
0(Tis)	4	4	0.953	5	3	0.443
I+II	24	22		25	21	
III+IV	22	24		20	26	
Lymphatic permeation						
Present	23	19	0.418	23	20	0.545
Absent	27	31		27	30	
Neurological invasion						
Present	21	17	0.410	22	27	0.317
Absent	29	33		28	23	
Vascular invasion						
Present	26	24	0.689	13	20	0.137
Absent	24	26		37	30	

^a^Not counting transverse colon and rectal cancer

^b^Not counting carcinoma in situ

Four carcinomas in situ in the FABP4 high and low expression group, respectively, and 5 and 3 carcinomas in situ in the FABP6 high and low expression group, respectively

### Correlations between serum levels of FABP4 and FABP6 and metabolic and anthropometric variables and other parameters

Spearman correlation coefficients for associations between FABP4 and FABP6 levels and metabolic and anthropometric variables and other parameters among cases and control participants are shown in Table 3. Among patients with CRC, FABP4 levels were positively associated with BMI, WHR, and TG (BMI: *r* = 0.277, *P* = 0.005; WHR: *r* = 0.182, *P* = 0.040; TG: *r* = 0.215, *P* = 0.013) and FABP6 levels were positively associated with BP (SBP: *r* = 0.248, *P* = 0.013; DBP: *r* = 0.291, *P* = 0.003). When restricted to the control group, we can merely find FABP4 levels were positively correlated with BMI and WHR (BMI: *r* = 0.227, *P* = 0.024; WHR: *r* = 0.179, *P* = 0.048). We did not observe any significant correlations of FABP4 and FABP6 levels with age, sex, FPG, TCH, HDL-C, LDL-C, CEA, and CA19-9 in the CRC and control groups. In addition, among patients with CRC, FABP4 levels were not significantly associated with BP, and FABP6 levels were not significantly associated with BMI, WHR, and TG. Similarly, among the control group, FABP6 levels were not significantly associated with BMI or WHR.

**Table 3:** Correlations between FABP4 and FABP6 levels and anthropometric variables and other parameters

Variable	CRC group				control group			
	FABP4		FABP6		FABP4		FABP6	
	*r*	*P*	*r*	*P*	*r*	*P*	*r*	*P*
Age	0.017	0.867	0.002	0.981	0.020	0.854	0.042	0.631
Sex	0.079	0.436	− 0.019	0.853	− 0.122	0.226	0.120	0.314
BMI	0.277	0.005	0.067	0.511	0.227	0.024	0.035	0.727
WHR	0.182	0.040	0.042	0.631	0.179	0.048	0.102	0.311
SBP	− 0.022	0.828	0.248	0.013	− 0.010	0.323	− 0.138	0.172
DBP	− 0.046	0.650	0.291	0.003	− 0.045	0.655	0.015	0.883
FPG	0.135	0.182	0.112	0.269	0.116	0.249	0.088	0.385
TG	0.215	0.013	0.091	0.295	0.139	0.150	− 0.001	0.988
TCH	0.044	0.662	− 0.020	0.842	0.041	0.693	0.126	0.147
LDL-C	0.020	0.842	− 0.005	0.957	0.121	0.299	0.049	0.627
HDL-C	− 0.076	0.381	− 0.046	0.652	− 0.108	0.309	0.071	0.502
CEA	0.082	0.417	− 0.085	0.399	0.096	0.376	0.047	0.652
CA19-9	− 0.106	0.298	0.117	0.154	0.134	0.185	0.142	0.146
FABP6	0.121	0.193	---	---	0.163	0.055	---	---

Correlation coefficients and *P* values were determined using Spearman correlation analysis. *BMI* body mass index, *SBP* systolic blood pressure, *DBP* diastolic blood pressure, *WHR* waist:hip ratio, *TCH* total cholesterol, *TG* triglyceride, *LDL-C* low-density lipoprotein cholesterol, *HDL-C* high-density lipoprotein cholesterol, *FPG* fasting plasma glucose, *FABP4* fatty acid-binding proteins 4, *FABP6* fatty acid-binding proteins 6

### Evaluation of risk for colorectal cancer

Binary logistic regression analysis was performed with or without CRC as the dependent variable, and BMI (< 25.0 = 0, ≥ 25.0 = 1), SBP (< 140 = 0, ≥ 140 = 1), DBP (< 90 = 0, ≥ 90 = 1), WHR (man ≤ 1.0/female ≤ 0.9 = 0; man > 1.0/female > 0.9 = 1, respectively), TCH (≤ 5.72 = 0, > 5.72 = 1), TG (≤ 1.70 = 0, > 1.70 = 1), LDL-C (≤ 3.37 = 0, > 3.37 = 1), HDL-C (≤ 1.04 = 0, > 1.04 = 1), FPG (< 6.1 = 0, ≥ 6.1 = 1), FABP4 (< 223.35 = 0, ≥ 223.35 = 1), FABP6 (< 347.26 = 0, ≥ 347.26 = 1), CEA (< 5.0 = 0, ≥ 5.0 = 1), CA19-9 (< 34 = 0, ≥ 34 = 1), and family history of CRC (no = 0, yes = 1) as independent variables. Univariate logistic regression analysis indicated that WHR, LDL-C, FABP4, FABP6, CEA, and family history of CRC were risk factors for CRC, and HDL-C was a protective factor. According to the results of univariate logistic regression and the previous studies about the impact of metabolic syndrome on CRC [23], we adjusted for WHR, SBP, DBP, LDL-C, HDL-C, CEA, and family history of CRC in multivariate logistic regression analysis (sample size 200), the results still showed that FABP4 and FABP6 are independent risk factors for CRC development (adjusted odds ratio 1.916; 95%CI 1.340–2.492; *P <* 0.001; adjusted odds ratio 2.162; 95%CI 1.046, 1.078); *P <* 0.001, respectively) (Table 4).

**Table 4:** Evaluation of risks for colorectal cancer

Variable	Unadjusted	*P* value	Adjusted	*P* value
	OR (95% CI)		OR (95% CI)	
BMI (kg/m2)	1.499 (0.710, 3.166)	0.289	---	---
WHR	2.138 (1.002, 4.584)	0.037	2.084 (0.949, 4.578)	0.047
SBP (mmHg)	1.286 (0.815, 1.641)	0.055	1.258 (0.565, 2.802)	0.575
DBP (mmHg)	1.372 (0.993, 1.707)	0.051	1.034 (0.856, 1.413)	0.064
TCH (mmol/L)	0.522 (0.209, 1.307)	0.165	---	---
TG (mmol/L)	0.927 (0.508, 1.692)	0.805	---	---
LDL-C (mmol/L)	4.301 (3.271, 5.432)	< 0.001	4.197 (3.144, 5.393)	< 0.001
HDL-C (mmol/L)	0.078 (0.028, 0.218)	< 0.001	0.124 (0.057, 0.274)	< 0.001
FPG (mmol/L)	0.979 (0.274, 3.493)	0.974	---	---
FABP4 (pg/ml)	2.141 (1.352, 3.074)	< 0.001	1.916 (1.340, 2.492)	< 0.001
FABP6 (pg/ml)	2.767 (1.517, 3.826)	< 0.001	2.162 (1.046,1.078)	< 0.001
CEA (ng/ml)	1.940 (1.038, 3.479)	0.026	1.713 (1.026, 3.236)	0.040
CA19-9 (U/ml)	3.516 (0.938, 6.186)	0.062	---	---
Family history of CRC	7.298 (5.210, 9.747)	< 0.001	5.119(3.940, 7.569)	< 0.001

Adjusted for WHR, SBP, DBP, LDL-C, HDL-C, CEA, family history of CRC; *OR* odd ratio, *BMI* body mass index, *SBP* systolic blood pressure, *DBP* diastolic blood pressure, *WHR* waist:hip ratio, *TCH* total cholesterol, *TG* triglyceride, *LDL-C* low-density lipoprotein cholesterol, *HDL-C* high-density lipoprotein cholesterol, *FPG* fasting plasma glucose, *FABP4* fatty acid-binding proteins 4, *FABP6* fatty acid-binding proteins 6, *CEA* carcinoembryoni, *CA19-9* carbohydrate antigen 19-9

### Marker validation

**Fig. 4:**
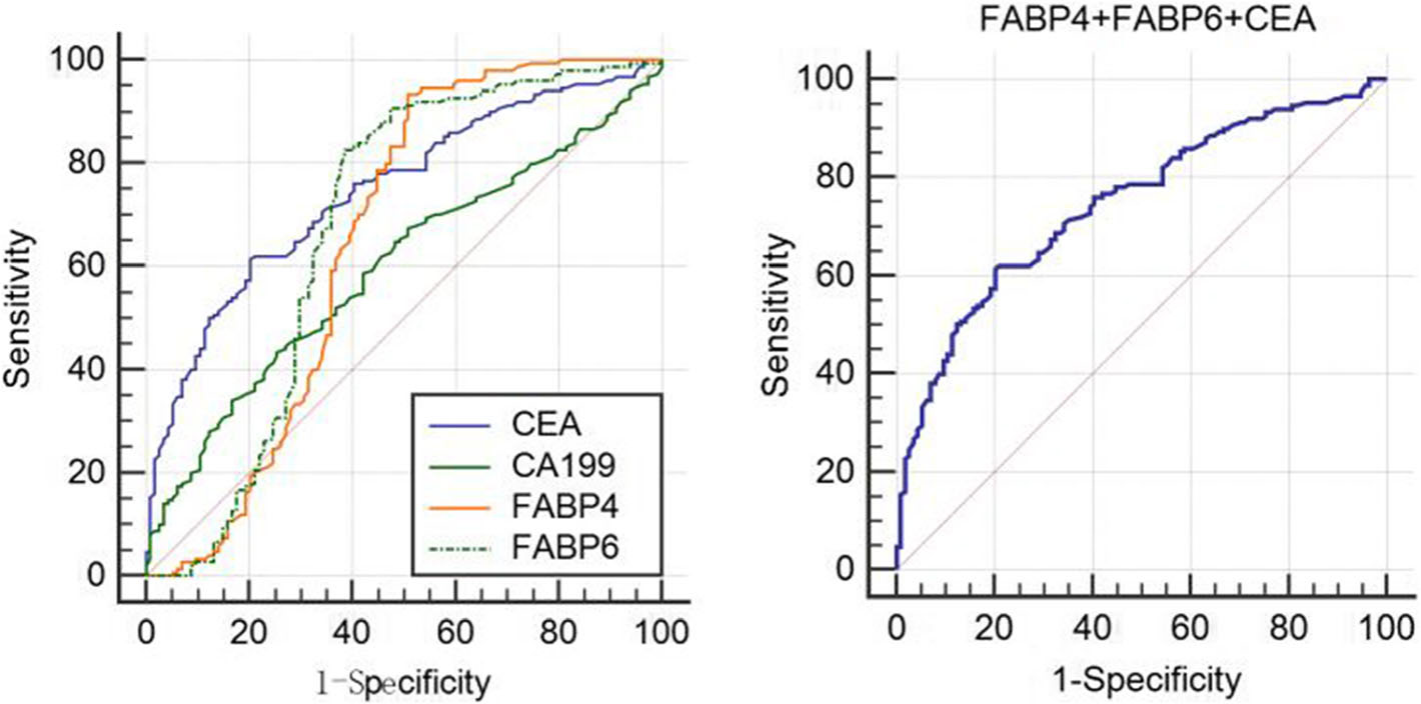
Receiver operating characteristics curve analysis using serum FABP4, FABP6,CEA, and CA199 in CRC, respectively (Left). Joint detection of FABP4, FABP, and CEA in CRC for discriminating CRC from normal subjects (Right). ROC curve analyses showed that the ROC curve areas for FABP4, FABP6, and CEA as well CA19-9 in CRC are 0.658 (95%CI 0.598–0.714), 0.683 (95%CI 0.624–0.738), 0.689 (95%CI 0.631–0.744), 0.592 (95%CI 0.531–0.651), respectively. The optimal sensitivity and specificity obtained by movement of the cutoff value of serum FABP4, which was 223.35 pg/ml, were 93.20% (95%CI 87.8–96.7) and 48.8% (95%CI 39.8–57.9) in discriminating CRC from the normal control. Similarly, the optimal sensitivity and specificity obtained by movement of the cutoff value of serum FABP6, which was 347.26 pg/ml, were 83.70% (95%CI 76.7–89.3) and 58.4% (95%CI 49.2–67.1) in discriminating CRC from the normal control. The optimal sensitivity and specificity obtained by movement of the cutoff value of serum CEA, which was 7.5 ng/ml, were 53.06% (95%CI 44.7–61.3) and 77.60% (95%CI 69.3–84.6) in discriminating CRC from the normal control, and the optimal sensitivity and specificity obtained by movement of the cutoff value of serum CA19-9, which was 14.24 U/ml, were 46.26% (95%CI 38.0–54.7) and 68.80% (95%CI 59.9–76.8) in discriminating CRC from the normal control. When combined detection of FABP4, FABP6, and CEA, the area of ROC curves is 0.746 (95% CI 0.689–0.798), and the optimal sensitivity and specificity were 61.33% (53.0–69.2) and 79.82% (71.3–86.8), respectively. Diagonal segments are produced by ties

To further verify the discriminating power of FABP4 and FABP6 identified for CRC diagnosis, serum levels of FABP4 and FABP6 were assessed on an independent group of 200 serum samples including 100 CRC patients and 100 normal controls. ROC curves analysis showed that the ROC curves areas for FABP4, FABP6, and CEA as well CA19-9 in CRC are 0.658 (95%CI 0.598–0.714), 0.683 (95%CI 0.624–0.738), 0.689 (95%CI 0.631–0.744), and 0.592 (95%CI 0.531–0.651), respectively. The optimal sensitivity and specificity obtained by movement of the cutoff value of serum FABP4, which was 223.35 pg/ml, were 93.20% (95%CI 87.8–96.7) and 48.8% (95%CI 39.8–57.9) in discriminating CRC from the normal control and positive predictive value (PPV) and negative positive predictive value (NPV) were 68.2% (95%CI *61.2*–74.5) and 85.9% (95%CI 75.6–93.0), respectively. Similarly, the optimal sensitivity and specificity obtained by movement of the cutoff value of serum FABP6, which was 347.26 pg/ml, were 83.70% (95%CI 76.7–89.3) and 58.4% (95%CI 49.2–67.1) in discriminating CRC from the normal control and PPV and NPV were 70.3% (95%CI 62.9–76.9) and 75.9% (95%CI 65.5–83.5), respectively. The optimal sensitivity and specificity obtained by movement of the cutoff value of serum CEA, which was 7.5 ng/ml, were 53.06% (95%CI 44.7–61.3) and 77.60% (95%CI 69.3–84.6) in discriminating CRC from the normal control, and PPV and NPV were 73.6% (95%CI 64.1–81.7) and 58.5% (95%CI 50.5–66.0); the optimal sensitivity and specificity obtained by movement of the cutoff value of serum CA19-9, which was 14.24 U/ml, were 46.26% (95%CI 38.0–54.7) and 68.80% (95%CI 59.9–76.8) in discriminating CRC from the normal control, and PPV and NPV were 63.6% (95%CI 53.7–72.6) and 52.1% (95%CI 44.2–59.9), respectively. When combined detection of FABP4, FABP6, and CEA, the area of ROC curves is 0.746 (95% CI 0.689–0.798), and the optimal sensitivity and specificity were 61.33% (53.0–69.2) and 79.82% (71.3–86.8), and PPV and NPV were 80.0% (95%CI 71.5–86.9) and 61.1% (95%CI 52.8–68.9), respectively, and the diagnostic efficiency was higher than any single index (*P* < 0.05) (Fig. 4).

## Discussion

The results showed that the mean serum levels of FABP4 and FABP6 in the CRC group were significantly higher than in the control group before surgery, and significantly decreased at 2 weeks after operation. Immunohistochemistry showed that FABP4 and FABP6 were mainly distributed in the cytoplasm of human colorectal tumor tissues, and only a small amount distributed in adjacent tissues. Western blot revealed that the protein expressions of FABP4 and FABP6 were significantly higher in tumor tissues than in adjacent tissues. Multivariate logistic regression analysis showed that the patients with higher serum FABP4 and FABP6 levels present an elevated risk of CRC independent of other confounding factors. In addition, we found the serum FABP4 and FABP6 levels could provide a potential biomarker to predict CRC, especially, combined detection of CEA with FABP4 and FABP6 could improve the diagnostic efficacy (sensitivity and specificity) of CRC. Therefore, the results of this study suggest that increased levels of FABP4 and FABP6 not only were strong risk factors for CRC but also could represent potential biomarkers for diagnosis of CRC in Chinese patients.

FABPs are structurally conserved intracellular lipid transporters that bind unesterified long-chain fatty acids and other ligands with nanomolar affinity and a molecular weight of approximately 15 kDa [24]. These transporters are abundantly expressed in most tissue cells and function by binding to lipid ligands. FABPs were originally described as intracellular proteins that affect intracellular energy metabolism, inflammatory immune responses, and signaling in certain diseases (e.g., obesity, diabetes, atherosclerosis) [25–27]. With the research of transgenic and gene knockout technology and the development of proteomics, FABPs have been found to be closely related to tumors. Therefore, making a deep research of the tumor-causing mechanism of FABPs and its impact on tumor cells will provide new ideas for blocking the occurrence and development of tumors.

It is well known that FABP4 is primarily expressed in adipocytes and macrophages. In recent years, studies have found that FABP4 may play an important role in metabolic syndrome and the pathogenesis of liver cancer caused by obesity [28]. Thompson et al. showed that the level of FABP4 is upregulated in a mouse model of obesity-induced hepatocellular carcinoma, and in vitro studies revealed that FABP4 promoted proliferation and migration of human hepatocellular carcinoma [29]. FABP6 is a cancer-associated protein that transports bile acids in ileal epithelial cells, and bile acids are known to play an important role in the development of CRC. In this study, we compared the serum levels of FABP4 and FABP6 in 100 CRC patients and 100 normal control subjects. The results showed that the serum levels of FABP4 and FABP6 in CRC patients were much higher than those in normal control subjects. In addition, after adjusting for potential confounding factors, logistic regression analysis showed that FABP4 and FABP6 were the independent risk factors for CRC, suggesting a close relationship between FABP4 and FABP6 levels and the development of CRC. To our knowledge, our study is the first clinical study in the literature to find increased levels of FABP4 and FABP6 are strong risk factors for CRC.

Ohmachi et al. revealed that tumors with high FABP6 expression were smaller in size, more often in the left colon and had shallower invasion into the bowel wall compared with those with low expression, and there was no significant difference in histologic type, lymph node, or liver metastasis, Dukes’ classification, and prognosis (13). However, we did not find a link between serum levels of FABP4 and FABP6 and the clinicopathologic features of CRC. The reasons why our findings are inconsistent with those of Ohmachi et al. may include race, sample size, and so on. Therefore, further verification is worth conducting in the future to elucidate association between expressions of FABP4, FABP6, and the clinicopathologic features of CRC.

Interestingly, in this study, immunohistochemistry and western blot analysis showed that FABP4 and FABP6 were mainly expressed in the cells from tumor tissues, and only a small amount distributed in adjacent tissues. Meanwhile, compared with preoperative levels, the serum levels of FABP4 and FABP6 at 2 weeks after surgery were significantly reduced. However, we found that the above decrease amplitude of FABP4 and FABP6 was merely 11.3% and 13.8%, respectively, both FABP4 and FABP6 levels in CRC group after surgery were still higher than those in the control group. These results suggested the causes of high serum levels of FABP4 and FABP6 in patients with CRC are complicated. Tumor-induced secretion may only be one of the reasons for the increase of FABP4 and FABP6 in peripheral serum. This may explain why the serum levels of FABP4 and FABP6 in CRC patients cannot be completely reversed after surgical removal of tumor tissue. However, the partial results of our study were inconsitent with previous studies. Shen et al. and Sayagués et al. explored the expression of FABP4 in patients with CRC by microarray analysis, the former studies showed that decreased FABP4 gene expression was identified from tumor samples compared with adjacent non-cancerous controls [19], the latter studies revealed that FABP4 expression levels are normal from primary tumors in CRC, whereas FABP4 expression is reduced of liver metastases from CRC patients [30]. We speculate that the possible reasons why our findings are paradoxical with other studies include difference in genetic differences among subjects, clinical characteristics of subjects (e.g., tumor stage, body fat percentage, BMI, nutritional status), and methodology. In fact, in the present study, we showed that FABP4 and FABP6 are independent risk factors for CRC development. Jin et al. deemed that FABP4 promotes EMT by the AKT/GSK3 β/Snail signaling pathway in cervical squamous cell carcinoma [31], and Thompson et al. believed FABP4 promoted proliferation and migration of human hepatocellular carcinoma [29]. These results might indirectly support our findings. Therefore, additional experiments will be required to confirm our results.

Ideal biomarkers should be highly differentiated from other lesions, such as cancer, normal lesions, or adenomas, and are continuously released into the lumen or circulation and disappear or reduce after the lesion is removed or treated. In our preliminary observational study (data unpublished), we found that serum concentrations of FABP4 and FABP6 increased before operation and gradually reduced postoperatively in patients with CRC, consistent with changes of serum levels of CEA and CA19-9 which are well known biomarkers of CRC. The present study further confirmed our previous finding. Thus, we consider that FABP4 and FABP6 may be suitable biomarkers for diagnosing relatively early CRC and/or assessing anticancer therapy. Regrettably, the ROC curves showed that the area under the curves (AUC) of each of the four indicators (FABP4, FABP6, CEA, and CA199) was lower than 0.7. Compared with CEA and CA199, FABP4 and FABP6 are more sensitive but less specific. CEA combined with FABP4 and FABP6 have higher diagnostic efficacy than any single indicator. Therefore, it is clinically possible to jointly detect FABP4 and FABP6 with CEA to improve the diagnosis rate of early CRC. Excitingly, the recent study reported by Long et al. showed that several genes involved in cellular energetic could be candidate for diagnostic, prognostic, and predictive biomarkers of CRC by high-throughput omics and statistical learning integration [32], and their results further supported our point of view.

A correlation analysis showed that FABP4 was positively associated with BMI and WHR among subjects both in CRC group and control group. Moreover, FABP4 was positively associated with TG and FABP6 levels were positively correlated with SBP and DBP in CRC group, respectively. In adipocytes, FABP4 activates hormone-sensitive lipase (HSL) to regulate lipolysis of adipocytes, and lipid metabolism disorder and chronic inflammatory response are two important characteristics of obesity. Studies [33, 34] have shown that knocking out the FABP4 gene in mouse adipocytes can reduce the expression of inflammatory factors in macrophages and the inflammatory response in adipose tissue, which can lead to obesity. These previous studies strengthened our findings that FABP4 was positively associated with BMI, WHR, and TG. As for FABP6, it played an important role in the transport of bile acids, and bile acids are involved in the pathogenesis of essential hypertension as an endogenous inhibitor of 11β-hydroxysteroid dehydrogenase [35].Thus, these findings can explain why FABP6 is related to SBP and DBP in our study.

In addition, in the present study, we revealed a significant increased OR of WHR, LDL-C, CEA, and CRC family history and a significant decreased OR of HLD-C in patients with CRC by univariate regression analysis, which is consistent with previous studies [36–38]. As we all know, CEA is currently the best characteristic serum tumor marker for screening for CRC and associated with prognosis and recurrence of CRC [39]. WHR and CRC family history are recognized risk factors for CRC. Aleksandrova et al. showed that LDL-C promotes proliferation of tumor cells in vitro by regulating apoptosis, and mitogen-activated protein kinase-dependent pathway plays an important role in the development of colorectal cancer [37]. A large European study found that high levels of plasma HDL-C significantly and independently protect subjects from colon cancer risk after adjusting for other confounding factors. Plasma HDL concentrations increased by 16 mg/dl, and colon cancer risk decreased by 22% [38].

The current molecular mechanism between FABP4 and FABP6 and the onset of CRC are unclear. Some scholars believed that there is a potential link between FABP4 and hyperlipidemia, hyperinsulinemia, and insulin resistance [40], which indirectly affects cancer cells by affecting these factors. Hotamisligil et al. found that in FABP4-deficient mice or knockout mouse models, the mice gained weight, had improved insulin resistance, and decreased total cholesterol and triglyceride levels [41], which further validated this view. Thompson et al. studied the high expression of FABP4 in human hepatocarcinoma models and animal models, and concluded that FABP4 can be synthesized and secreted by hepatocytes and hepatoma cells [29]; thus, the authors speculate that CRC cells may synthesize and secrete FABP4 like liver cancer cells. In addition, studies have reported that FABP4 affects cell growth and promotes tumor cell metastasis by carrying fatty acid transport energy or through the MAPK pathway [7, 42]. As for FABP6, Venturi et al. believed that in the early stage of CRC, FABP6 transports bile acids, and excessive bile acids infiltrate epithelial cells, induce apoptosis, and indirectly cause DNA damage, leading to impaired gene regulation of colonic epithelial cells [43].

Notwithstanding, this study also has several shortcomings, such as the relatively small sample size; the levels of FABP4 and FABP6 were measured only 2 weeks after surgery; a longer-term dynamic follow-up measurement on patient’s treatment, treatment effect, survival time, and FABPs levels were not performed; without using the more sensitive PCR methods to determine the mRNA expressions of FABP4 and FABP6. Thus, the relationship between FABP4 and FABP6 expression levels and treatment response and prognosis survival in CRC patients has remained unknown.

In conclusion, this study found that serum levels of FABP4 and FABP6 were significantly increased in patients with CRC, and the increased expressions of FABP4 and FABP6 were associated with the development of CRC. We also revealed that serum FABP4 and FABP6 may represent potential biomarkers for CRC diagnosis in Chinese patients. Combined detection of CEA with FABP4 and FABP6 could improve the diagnostic efficacy of CRC. However, this study is still unable to determine the causal relationship between FABP4 and FABP6 and the onset of CRC, which is to be confirmed by in vitro cell studies and animal experiments.

## Data Availability

The analyzed data sets generated during the study are available from the corresponding author on reasonable request. Inquiries for data access may be sent to the following e-mail address: chmw1@163.com.

## Abbreviations

BMI: Body mass index
CA199: Carbohydrate antigen 19-9
CEA: Carcinoembryonic antigen
CRC: Colorectal cancer
DAB: Diaminobenzidine
DBP: Diastolic blood pressure
EDTA: Ethylenediaminetetraacetic acid
ELISA: Enzyme-linked immunosorbent assay
FABP4: Fatty acid-binding protein 4
FABP6: Fatty acid-binding protein 6
HDL-C: High-density lipoprotein cholesterol
LDL-C: Low-density lipoprotein cholesterol
PBS: Phosphate-buffered saline
RIPA: Radioimmunoprecipitation assay
ROC: Receiver operator characteristic
SBP: Systolic blood pressure
SDS-PAGE: Sodium dodecyl sulphate polyacrylamide gel electrophoresis
TC: Total cholesterol
TG: Triacylglycerol
WC: Waist circumference
WHR: Waist-to-hip ratio

## References

[1] Bray F, Ferlay J, Soerjomataram I, Siegel RL, Torre LA, Jemal A. Global cancer statistics 2018:GLOBOCAN estimates of incidence and mortality worldwide for 36 cancers in 185 countries. CA Cancer J Clin. 2018. 10.3322/caac.21492 30207593

[2] Siegel R, DeSantis C, Jemal A (2014). Colorectal cancer statistics. CA Cancer J Clin.

[3] Siegel RL, Miller KD, Jemal A (2016). Cancer statistics. CA Cancer J Clin.

[4] Center MM, Jemal A, Smith RA, Ward E (2009). Worldwide variations in colorectal cancer. CA Cancer J Clin.

[5] Storch J, Thumser AE (2010). Tissue specific functions in the fatty acid-binding protein family. J Biol Chem.

[6] Kralisch S, Fasshauer M (2013). Adipocyte fatty acid binding protein: a novel adipokine involved in the pathogenesis of metabolic and vascular disease?. Diabetologia..

[7] Nieman KM, Kenny HA, Penicka CV, Ladanyi A, Buell-Gutbrod R, Zillhardt MR (2011). Adipocytes promote ovarian cancer metastasis and provide energy for rapid tumor growth. Nat Med.

[8] ImrayCH RS, Al D (1992). Faecal unconjugated bile acids in patients with colorectal cancer or polyps. Gut.

[9] Korpela JT, Adlercreutz H, Turunen MJ (1988). Fecal free and conjugated bile acids and neutral sterols in vegetarians, omnivores, and patients with colorectal cancer. Scand J Gastroenterol.

[10] Hill MJ, Melville DM, Lennard-Jones JE, Neale K, Ritchie JK (1987). Faecal bile acids, dysplasia, and carcinoma in ulcerative colitis. Lancet.

[11] Kurtz WJ, Leuschner U (1983). Bile acids in patients suffering from colorectal carcinoma Ha pilot study. Tokai J Exp Clin Med.

[12] Fujii H, Nomura M, Kanda T, Amano O, Iseki S, Hatakeyama K (1993). Cloning of a cDNA encoding rat intestinal 15 kDa protein and its tissue distribution. Biochem Biophys Res Commun.

[13] Ohmachi T, Inoue H, Mimori K, Tanaka F, Sasaki A, Kanda T (2006). Fatty acid binding protein 6 is overexpressed in colorectal cancer. Clin Cancer Res.

[14] Jing C, Beesley C, Foster CS, Rudland PS, Fujii H, Ono T, Chen H (2000). Identification of the messenger RNA for human cutaneous fatty acid-binding protein as a metastasis inducer. Cancer Res.

[15] Guaita-Esteruelas S, Bosquet A, Saavedra P, Gumà J, Girona J, Lam EWF (2017). Exogenous FABP4 increases breast cancer cell proliferation and activates the expression of fatty acid transport proteins. Mol Carcinog.

[16] Das R, Hammamieh R, Neill R, Jett M (2001). Expression pattern of fatty acid-binding proteins in human normal and cancer prostate cells and tissues. Clin Cancer Res.

[17] Hashimoto T, Kusakabe T, Sugino T, Fukuda T, Watanabe K, Sato Y (2004). Expression of heart-type fatty acid-binding protein in human gastric carcinoma and its association with tumor aggressiveness, metastasis and poor prognosis. Pathobiology.

[18] Bao Z, Malki MI, Forootan SS, Adamson J, Forootan FS, Chen D (2013). A novel cutaneous fatty acid-binding protein-related signaling pathway leading to malignant progression in prostate cancer cells. Genes Cancer.

[19] Shen X, Yue M, Meng F, Zhu J, Zhu X, Jiang Y (2016). Microarray analysis of differentially- expressed genes and linker genes associated with the molecular mechanism of colorectal cancer. Oncol Lett.

[20] Zhao Dongmei, Ma Yanying, Li Xu, Lu Xiaoyu (2019). microRNA‐211 promotes invasion and migration of colorectal cancer cells by targeting FABP4 via PPARγ. Journal of Cellular Physiology.

[21] Chen M, Wang Y, Li Y, Zhao L, Ye S, Wang S (2016). Association of plasma visfatin with risk of colorectal cancer: an observational study of Chinese patients. Asia-Pacific Journal of Clinical Oncology.

[22] Perkins NJ, Schisterman EF (2006). The inconsistency of “optimal” cut-points obtained using two criteria based on the receiver operating characteristic curve. Am J Epidemiol.

[23] Ulaganathan V, Kandiah M, Shariff ZM (2018). A case-control study of the association between metabolic syndrome and colorectal cancer: a comparison of International Diabetes Federation, National Cholesterol Education Program Adults Treatment Panel III, and World Health Organization definitions. J Gastrointest Oncol.

[24] Liu RZ, Denovan-Wright EM, Wright JM (2003). Structure, linkage mapping and expression of the heart-type fatty acid-binding protein gene (fabp3) from zebrafish (*Danio rerio*). Eur J Biochem.

[25] Parra S, Cabre A, Marimon F, Ferre R (2014). Circulating FABP4 is a marker of metabolic and cardiovascular risk in SLE patients. Lupus..

[26] Bingold TM, Franck K, Holzer K, Zacharowski K (2015). Intestinal fatty acid binding 2. Protein: a sensitive marker in abdominal surgery and abdominal infection. Surg Infect (Larchmt).

[27] Cramer G, Bakker J, Gommans F (2014). Relation of highly sensitive cardiactroponin T in hypertrophic cardiomyopathy to left ventricular mass and cardiovascular risk. Am J Cardiol.

[28] El-Serag HB, Kanwal F (2014). Epidemiology of hepatocellular carcinoma in the United States: where are we? Where do we go?. Hepatology..

[29] Thompson KJ, Austin RG, Nazari SS, Gersin KS, Iannitti DA, McKillop IH (2017). Altered fatty acid-binding protein 4 (FABP4) expression and function in human and animal models of hepatocellular carcinoma. Liver Int.

[30] Sayagués JM, Corchete LA, Gutiérrez ML, Sarasquete ME, Del Mar AM, Bengoechea O (2016). Genomic characterization of liver metastases from colorectal cancer patients. Oncotarget..

[31] Jin Jiangbo, Zhang Ziyu, Zhang Song, Chen Xinyu, Chen Zhen, Hu Ping, Wang Jianbin, Xie Caifeng (2018). Fatty acid binding protein 4 promotes epithelial-mesenchymal transition in cervical squamous cell carcinoma through AKT/GSK3β/Snail signaling pathway. Molecular and Cellular Endocrinology.

[32] Long NP, Park S, Anh NH, Nghi TD, Yoon SJ, Park JH (2019). High-throughput Omics and statistical learning integration for the discovery and validation of novel diagnostic signatures in colorectal cancer. Int J Mol Sci.

[33] Furuhashi M, Fucho R, Gorgun CZ, Tuncman G, Cao H, Hotamisligil GS (2008). Adipocyte/macrophage fatty acid binding proteins contribute to metabolic deterioration through actions in both macrophages and adipocytes in mice. J Clin Invest.

[34] Bernlohr DA, Coe NR, Simpson MA, Hertzel AV. Regulation of gene expression in adipose cells by polyunsaturated fatty acids. Adv Exp Med Biol. 1997;422. 10.1007/978-1-4757-2670-1_12 9361822

[35] Morris DJ, Souness GW (1996). Endogenous 11β-hydroxysteroid dehydrogenase inhibitors and their role in glucocorticoid Na+ retention and hypertension. Endocr Res.

[36] Ulaganathan V, Kandiah M, Shariff ZM (2018). A case-control study on the association of abdominal obesity and hypercholesterolemia with the risk of colorectal cancer. J Carcinog.

[37] Aleksandrova K, Jenab M, Bueno-de-Mesquita HB, Fedirko V, Kaaks R, Lukanova A (2014). Biomarker patterns of inflammatory and metabolic pathways are associated with risk of colorectal cancer: results from the European Prospective Investigation into Cancer and Nutrition (EPIC). Eur J Epidemiol.

[38] Marotta T, Viola S, Ferrara F, Ferrara LA (2007). Improvement of cardiovascular risk profile in an elderly population of low social level: the ICON (Improving Cardiovascular Risk Profile in Older Neapolitans) Study. J Hum Hypertens.

[39] Huang L, Fang J, Wu J, Zhou X, Wei H (2018). Prognostic value of combining preoperative serum tumor markers and peripheral blood routine indexes in patients with colorectal cancer. Zhonghua Wei Chang Wai Ke Za Zhi.

[40] Uysal KT, Scheja L, Wiesbrock SM, Bonner-Weir S, Hotamisligil GS (2000). Improved glucose and lipid metabolism in genetically obese mice lacking aP2. Endocrinology.

[41] Hotamisligil GS, Johnson RS, Distel RJ, Ellis R, Papaioannou VE, Spiegelman BM (1996). Uncoupling of obesity from insulin resistance through a targeted mutation in aP2, the adipocyte fatty acid binding protein. Science.

[42] Lee D, Wada K, Taniguchi Y (2014). Expression of fatty acid binding protein 4 is involved in the cell growth of oral squamous cell carcinoma. Oncology Reports.

[43] Venturi M, Hambly RJ, Glinghammar B, Rafter JJ, Rowland IR (1997). Genotoxic activity in human faecal water and the role of bile acids: a study using the alkaline comet assay. Carcinogenesis.

